# A triple-drug nanotherapy to target breast cancer cells, cancer stem cells, and tumor vasculature

**DOI:** 10.1038/s41419-020-03308-w

**Published:** 2021-01-04

**Authors:** Sara El-Sahli, Khang Hua, Andrew Sulaiman, Jason Chambers, Li Li, Eliya Farah, Sarah McGarry, Dan Liu, Peiyong Zheng, Seung-Hwan Lee, Jiefeng Cui, Marc Ekker, Marceline Côté, Tommy Alain, Xuguang Li, Vanessa M. D’Costa, Lisheng Wang, Suresh Gadde

**Affiliations:** 1 Department of Biochemistry, Microbiology and Immunology, Faculty of Medicine, University of Ottawa, 451 Smyth Road, Ottawa, ON K1H 8M5 Canada; 2 Department of Biology, Faculty of Science, University of Ottawa, 30 Marie Curie Ottawa, Ottawa, ON K1N 6N5 Canada; 3 Department of Genetics, School of Basic Medicine, Qiqihar Medical University, No.333 Bukui North Street, Jianhua District, 161006 Qiqihar, Heilongjiang People’s Republic of China; 4 Institute of Digestive Diseases, Longhua Hospital, Shanghai University of Traditional Chinese Medicine, 725 South Wanping Road, 200032 Shanghai, People’s Republic of China; 5 Liver Cancer Institute, Zhongshan Hospital, Fudan University & Key Laboratory of Carcinogenesis and Cancer Invasion, Ministry of Education, 136 Xue Yuan Road, 200032 Shanghai, People’s Republic of China; 6 Centre for Biologics Evaluation, Biologics and Genetic Therapies Directorate, Health Canada, Sir Frederick G. Banting Research Centre, 251 Sir Frederick G. Banting, Ottawa, ON K1Y 0M1 Canada; 7 Centre for Infection, Immunity and Inflammation, University of Ottawa, 451 Smyth Road, Ottawa, ON K1H 8M5 Canada; 8 Ottawa Institute of Systems Biology, University of Ottawa, 451 Smyth Road, Ottawa, ON K1H 8M5 Canada

**Keywords:** Breast cancer, Cancer stem cells

## Abstract

Triple-negative breast cancer (TNBC) is the most aggressive subtype of breast cancer, accounting for the majority of breast cancer-related death. Due to the lack of specific therapeutic targets, chemotherapeutic agents (e.g., paclitaxel) remain the mainstay of systemic treatment, but enrich a subpopulation of cells with tumor-initiating capacity and stem-like characteristics called cancer stem cells (CSCs); thus development of a new and effective strategy for TNBC treatment is an unmet medical need. Cancer nanomedicine has transformed the landscape of cancer drug development, allowing for a high therapeutic index. In this study, we developed a new therapy by co-encapsulating clinically approved drugs, such as paclitaxel, verteporfin, and combretastatin (CA4) in polymer-lipid hybrid nanoparticles (NPs) made of FDA-approved biomaterials. Verteporfin is a drug used in the treatment of macular degeneration and has recently been found to inhibit the Hippo/YAP (Yes-associated protein) pathway, which is known to promote the progression of breast cancer and the development of CSCs. CA4 is a vascular disrupting agent and has been tested in phase II/III of clinical trials. We found that our new three drug-NP not only effectively inhibited TNBC cell viability and cell migration, but also significantly diminished paclitaxel-induced and/or CA4-induced CSC enrichment in TNBC cells, partially through inhibiting the upregulated Hippo/YAP signaling. Combination of verteporfin and CA4 was also more effective in suppressing angiogenesis in an in vivo zebrafish model than single drug alone. The efficacy and application potential of our triple drug-NPs were further assessed by using clinically relevant patient-derived xenograft (PDX) models. Triple drug-NP effectively inhibited the viability of PDX organotypic slide cultures ex vivo and stopped the growth of PDX tumors in vivo. This study developed an approach capable of simultaneously inhibiting bulk cancer cells, CSCs, and angiogenesis.

## Introduction

Breast cancer is the most common cancer among women and the second leading cause of cancer-related death in women^1^. Triple-negative breast cancer (TNBC), a subset of breast cancer, accounts for the majority of breast cancer death due to the lack of specific treatment targets, as TNBC does not express estrogen receptor, progesterone receptor, and human epidermal growth factor receptor-2^2^. As such, chemotherapeutic agents (e.g., paclitaxel) remain the current first-line therapy for the patients with TNBC. While chemotherapeutic drugs effectively kill bulk cancer cells, ample evidence has demonstrated that chemotherapy enriches a subpopulation of cells known as cancer stem cells (CSCs), capable of initiating new tumors^3^. Breast CSCs are known to resist treatments and are key to cancer progression, recurrence, and metastasis^4^. Indeed, multiple reports have revealed the enrichment of CSCs in TNBC cell lines and associate it with the aggressive nature of TNBC^4,5^.

The Hippo signaling pathway is implicated in tumorigenesis and CSC enrichment. Its major downstream effector, Yes-associated protein (YAP), is known to play a significant role in the maintenance of a stem-like phenotype^6^. Furthermore, YAP expression correlates with the metastatic potential of breast cancer, and is a well-known driver in CSCs^6^. Verteporfin, an FDA-approved drug used in the treatment of macular degeneration, was found to inhibit cancer cell proliferation by inducing the cytoplasmic sequestration of YAP^7^.

Tumor growth and metastasis is also dependent on angiogenesis, the process by which new blood vessels are generated from existing ones^8^. The vascular disrupting agent, combretastatin A4 (CA4) is currently in phase II/III clinical trials and has been shown to be an effective antiangiogenesis agent^9^. In addition, CA4 is a tubulin binding chemotherapeutic agent and is shown to suppress a variety of cancers^9,10^. However, the effect of CA4 on CSC remains poorly explored. Given that TNBC aggression is a multifaceted process without specific targets, we sought to develop a new therapeutic strategy capable of simultaneously inhibiting bulk tumor cells, CSCs, and angiogenesis by the combination of paclitaxel, verteporfin, and CA4.

While combinational therapies have several advantages, dissimilar pharmacokinetics and off-target toxicities arising from the drug combinations hamper potential clinical application^11^. To reduce free drug toxicity, improve their pharmacokinetics, and induce an overlap in pharmacological profiles of the drugs, we employed a nanomedicine-based multidrug delivery platform^12^. Nanomedicine in cancer treatment has allowed for a better drug delivery with higher therapeutic index by virtue of features, such as improved circulation in blood, reduced off-target toxicity, and higher drug accumulation in the tumor^12,13^. This is thought to occur through the enhanced permeability and retention (EPR) effect, where molecules exceeding a certain size accumulate in tumor tissue due to the aberrant blood vasculature and the lack of lymphatic drainage within the tumor^14^.

Clinical translation of therapeutics tested in vivo is often limited by the use of cell line-based tumor models due to their artificial nature, which has created a gap between preclinical and clinical research^15^. Patient-derived xenograft (PDX) tumors, obtained from the patients and surgically implanted in mice, are capable of simulating in vivo patient tumors and display a strong retrospective correlation with actual patient responses^15,16^. In this study, we use an ex vivo and in vivo PDX tumor models that retain original tumor architecture, composition, vasculature, and heterogeneity in addition to in vitro TNBC cell line and in vivo zebrafish model.

We showed that paclitaxel and CA4 inhibited the viability of bulk TNBC cells, while enriching CSCs and upregulating oncogenic YAP signal, both of which can be effectively counteracted by verteporfin. As a result, triple drug-nanoparticle (NP) not only inhibited bulk TNBC cell viability, cell migration, but also suppressed CSC enrichment. Using an in vivo zebrafish model, we found that combination of CA4 and verteporfin more effectively abrogated angiogenesis than single drug alone. Furthermore, triple drug-NP effectively inhibited viability of PDX organotypic cultures ex vivo and disrupted the growth of TNBC PDX tumors in vivo. Given that the drugs and nanomaterials used in this report have been proved safe and employed in the clinic, this new nanomedicine-based approach tested in clinically relevant PDX models may lead to an effective treatment for TNBC patients.

## Results

### Nanoparticle synthesis and characterization

Lipid-polymer hybrid NP were synthesized using FDA-approved, biodegradable, and biocompatible poly (lactic-co-glycolic acid) (PLGA), polyethylene glycol (PEG) polymers, and encapsulated with single drug or triple drugs (paclitaxel, verteporfin, and CA4). All formulations of drug-NPs had a spherical morphology with surface charge of −5 to −50 mV and an average range of 100–150 nm in size. All NPs were incubated with fetal bovine serum (FBS) to assess stability; NP sizes were then measured and found to be stable in serum (Fig. S1A–E). The release rates of three drugs from the encapsulated NPs were also assessed (Fig. S2).

### Combination of verteporfin-NP, paclitaxel-NP, and/or combretastatin-NP suppressed both bulk TNBC cells and cancer stem cells

**Fig. 1:**
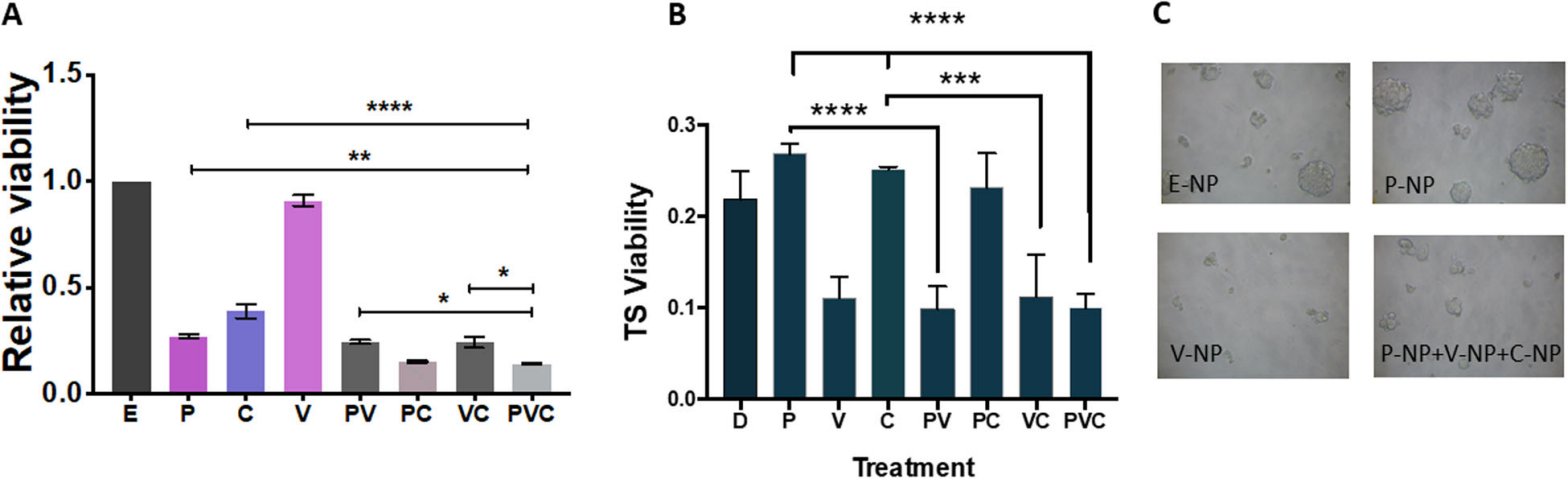
Combination of verteporfin-NP, paclitaxel-NP, and/or combretastatin-NP suppressed both bulk tumor cells and cancer stem cells. **A** The viability of TNBC MDA-MB-231 cells was determined by MTT assay after 120-hr treatment with E-NP (empty-nanoparticle), P-NP (paclitaxel-NP, 5 nM), V-NP (verteporfin-NP, 250 nM), C-NP (combretastatin-NP, 5 nM), or different combinations. **B** Tumorsphere (TS) formation, an in vitro assay indicating cancer stem cells (CSCs) after different treatments with drug-NPs. MDA-MB-231 cells overexpressing E-cadherin were grown in low attachment and serum-free conditions, and treated for 120 hrs as described in **A**, followed by MTT viability assay. **C** Representative photographs of TS shown in **B**. Data represent means ± SD, *P < 0.05, **P < 0.01, ****P < 0.001.

A viability assay was conducted to determine the effect of drugs-NP on TNBC MDA-MB-231 bulk cells in vitro. Results showed that paclitaxel-NP and CA4-NP in combination was more significant in decreasing bulk cell viability than each drug-NP alone, while verteporfin-NP exhibited minimal effect on bulk cells (Fig. 1A). The combination of the three drug-NP treatment also inhibited TNBC cell viability of the SUM149 cell line (Fig. S3).

Since breast CSCs are known to be key in cancer progression, metastasis, and recurrence^5,17^, targeting the CSC population with tumor-initiating capacity has been considered an effective strategy for successful treatment. To determine the effect of the NP-encapsulated drug treatment on CSCs, we carried out an in vitro tumorsphere assay, which is a method used commonly in cancer research to assess the function of CSCs in a 3D suspension culture^18^. Epithelial-like MDA-MB-231 cells (overexpressing E-cadherin) were grown in non-adherent and serum-free conditions, in which only CSC-like cells could survive and grow^18^. NP encapsulating drugs were then added to the cultures. After incubation for 120 hrs, tumorspheres were photographed and viability was assessed to determine CSC enrichment after different treatments. While paclitaxel-NP and CA4-NP inhibited bulk TNBC cells, they either enhanced CSC enrichment or did not exhibit inhibitory effects on CSCs (Fig. 1B, C). In contrast, although verteporfin-NP did not significantly inhibit viability of bulk TNBC cells, it markedly suppressed CSCs. Furthermore, verteporfin-NP in combination with paclitaxel-NP and/or CA4-NP significantly suppressed both bulk cells and CSCs (Fig. 1A–C). Consistently, flow cytometric analysis showed that the frequencies of apoptotic CSCs (CD44^+^ CD24^−^ Annexin-V^+^ 7AAD^−^) in TNBC cells were significantly increased after treatment with verteporfin alone or verteporfin in combination with paclitaxel and CA4. In contrast, paclitaxel and/or CA4 treatments did not show significant changes (Fig. S4).

These data suggest that three drug-NPs in combination not only suppress TNBC bulk tumor cells, but also inhibit CSCs, which cannot be achieved by either paclitaxel-NP nor CA4-NP alone. Verteporfin was essential to suppress the CSC enrichment and as such, the combination of three drug-NPs is required to suppress both bulk and CSCs for the development of an effective treatment for TNBC.

To evaluate the in vitro toxicity, non-transformed epithelial mammary cells (MCF-10A) were treated with P-NP, C-NP, V-NP, and different combinations. While drug-NPs significantly reduced cancer cell viability (Fig. 1A), they exhibited little effect on these non-transformed epithelial mammary cells (Fig. S5A).

### Verteporfin-NP effectively inhibits the migration of TNBC cells in vitro

**Fig. 2:**
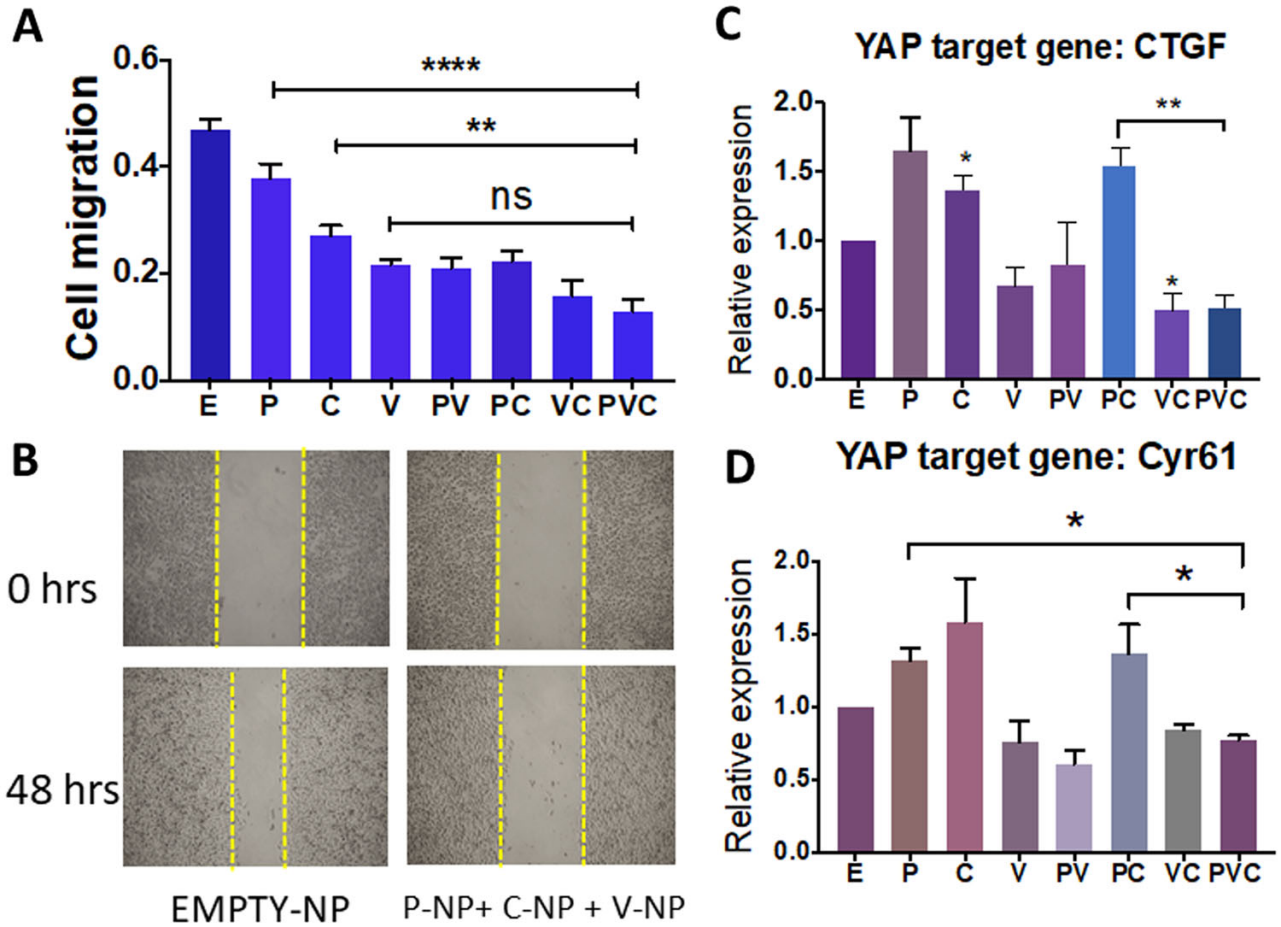
Verteporfin-NP more effectively inhibits the migration of TNBC cells and fully suppresses YAP target genes upregulated by paclitaxel-NP and/or combretastatin-NP. **A** TNBC MDA-MB-231 cells were grown to confluence, treated with mitomycin to inhibit cell proliferation, and then scratched and quantified for migration after incubation with E (empty-NP), 5 nM P (paclitaxel-NP), 250 nM V (verteporfin-NP), 5 nM C (combretastatin-NP), or different combinations for 48 hrs. **B** Representative images of A. **C**, **D** RT-qPCR analysis of changes of YAP target gene in MDA-MB-231 cells after 24 -hr of treatment with E (empty-NP), 25 nM P (paclitaxel-NP), 1.25 µM V (verteporfin-NP), 25 nM C (combretastatin-NP), and different combinations. Data represent means ± SEM, *P < 0.05, **P < 0.01, ****P < 0.0001.

Since cell migration has been attributed to cancer metastasis^19,20^, we assessed the effect of drug-NPs in combination on TNBC cell migration. An in vitro scratch assay was performed where TNBC MDA-MB-231 cells were exposed to mitomycin to stop cell proliferation, followed by a scratch. While verteporfin-NP was inadequate at inhibiting the viability of TNBC bulk cells (as shown in Fig. 1A), it effectively halted cell migration approximately two-fold greater than empty-NP control after a 48-hr treatment (Fig. 2A, B). Moreover, the three drug-NPs in combination suppressed migration effectively, highlighting the efficacy of verteporfin-NP and the three drug-NPs in halting the migration of TNBC cells in addition to promoting CSC apoptosis (shown in Fig. S4).

### Verteporfin-NP fully suppresses YAP target genes upregulated by paclitaxel-NP and/or combretastatin-NP

Of the many signaling pathways, YAP has recently been identified as an oncogene in several cancers, including TNBC^21^, and is closely associated with CSCs and poor prognosis in cancer patients^22^. Since paclitaxel-NP and CA4-NP treatment enriched CSCs, we asked whether the YAP signaling pathway was upregulated after treatment with these two drugs, and whether V-NP could abolish their upregulation of YAP activity. We assessed the effect of single drug-NP, and double or triple drug-NPs in different combinations on CSC-associated YAP target genes using RT-qPCR. While paclitaxel-NP and CA4-NP upregulated key YAP target genes, verteporfin-NP downregulated them. Importantly, triple drug-NPs in combination was able to suppress the YAP target genes upregulated by paclitaxel-NP and CA4-NP (Fig. 2C, D). These results suggest that suppression of YAP signal by verteporfin-NP in the triple drug-NP combination may contribute to the CSC inhibition (i.e., suppressing tumorsphere formation and promoting CSC apoptosis) as shown in Fig. 1 and Fig. S4.

### Drug combination effectively inhibits angiogenesis in an in vivo zebrafish model

**Fig. 3:**
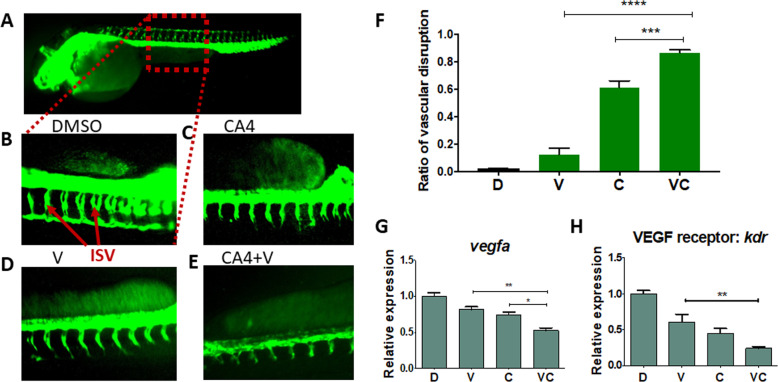
Verteporfin in combination with combretastatin more effectively inhibits vasculature in transgenic zebrafish (Tg, fli:eGFP) embryos. The zebrafish embryos at 8-hrs post fertilization were treated with drugs for 48 hrs. **A** Healthy zebrafish embryo taken at 8× magnification. Intersegmental vessel (ISV) phenotypes in a sectional view of: **B** DMSO; **C** CA4, 5 nM; **D** V, verteporfin, 250 nM; **E** V + CA4. **F** Graphical summary showing the ISV disruption ratio of B–E. Data are expressed as mean ± SEM, *n* = 5 embryos. **G**, **H** RT-qPCR analysis of vascular endothelial growth factor A (*VEGFA*, an angiogenesis gene) and VEGF receptor *kdr* in zebrafish embryos after treatments. Data are expressed as mean ± SEM, *n* = 4 embryos. *P < 0.05, **P < 0.01, ****P < 0.0001.

We further determined the effect of drug combinations on angiogenesis, the process by which new blood vessels are generated from the existing ones to promote tumorigenesis and tumor metastasis^8^. The zebrafish embryo model is the most effective way to study in vivo angiogenesis and is considered to be comparable to angiogenesis occurring in the clinic^23^. We used a transgenic fluorescent zebrafish, Tg(fli-GFP), which has GFP-expressing endothelial cells (lining the inside of blood vessel in the body) under the control of fli1 promoter to allow the visualization of blood vessels^24^. CA4 has been previously shown to inhibit angiogenesis in zebrafish^9^, we sought to determine the effect of verterporfin and CA4 in combination on angiogenesis. Zebrafish embryos at 8 hrs post fertilization were treated with drugs for 48 hrs. Since paclitaxel at the clinically relevant concentration had no effect on blood vessel formation (Fig. S6), it was not further tested in combinations. Consistent with literature, CA4 significantly inhibited angiogenesis in zebrafish as indicated by shortened/misshapen intersegmental vessels in comparison to the control (Fig. 3A–C). More significantly, combination of verteporfin and CA4 inhibited angiogenesis ~1.5-fold and 5-fold more than single drug alone, respectively (Fig. 3D–F).

To understand the mechanism underlying the antiangiogenic effect of the combination therapy, we analyzed angiogenesis-associated genes in zebrafish embryos and found a significant reduction in vascular endothelial growth factor A (*VEGFA*) and the VEGF receptor after treatment with verteporfin and CA4. Both drugs in combination suppressed these gene expressions more than single drug alone in vivo (Fig. 3G, H).

### Verteporfin also inhibits HIF-1α activity and the angiogenetic gene VEGF in vitro in human TNBC cells

**Fig. 4:**
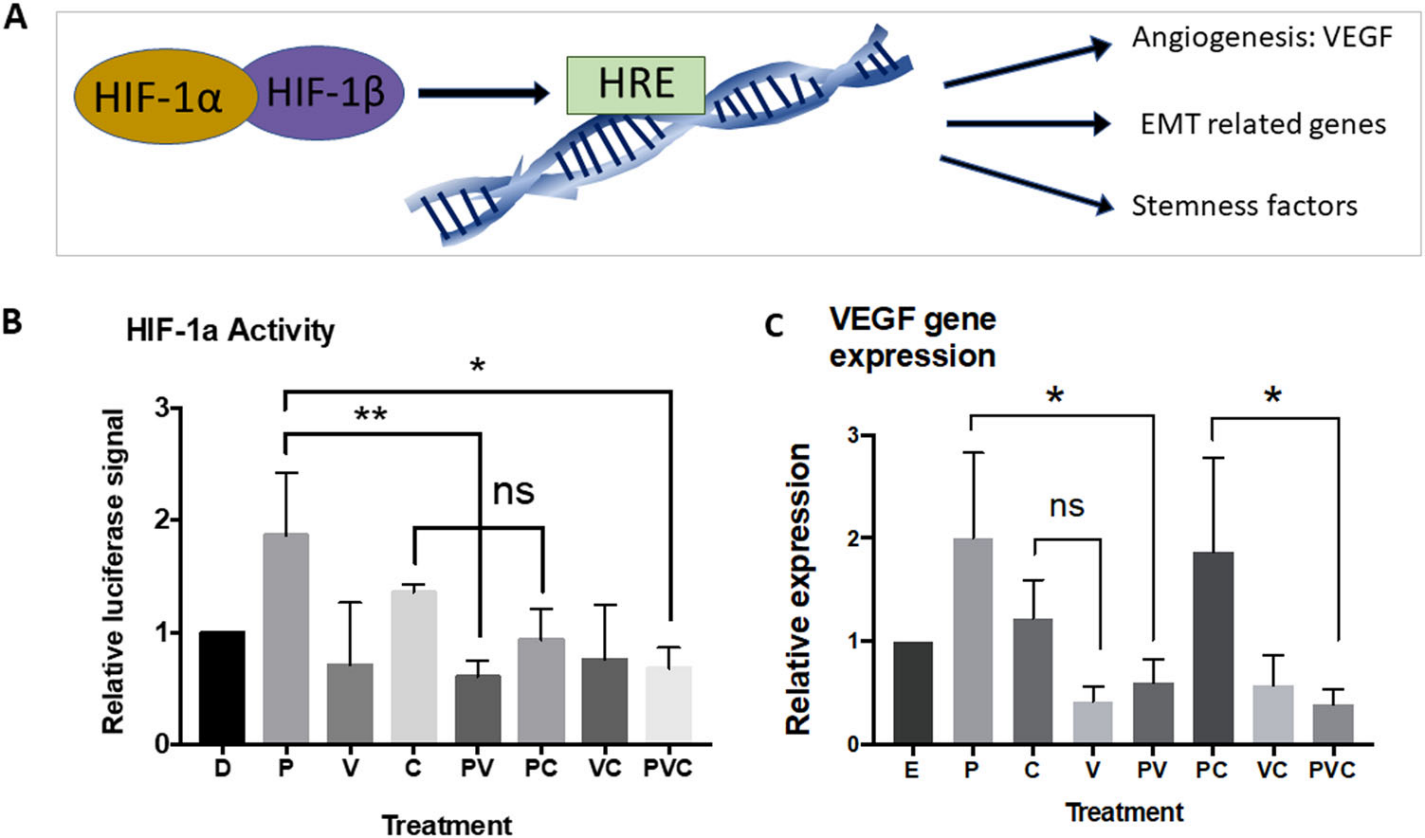
Verteporfin-NP also inhibits angiogenesis-associated genes in vitro in human TNBC cells. **A** Schematic showing that HIF-1α is a master regulator in angiogenesis and other tumorigenic processes. **B** Luciferase reporter activity of HIF-1α activities in MDA-MB-231 cells treated with control DMSO, paclitaxel (25 nM), verteporfin (1.25 µM), CA4 (25 nM), and different combinations for 24 hrs. **C** RT-qPCR analysis showing the changes of VEGF gene expression in MDA-MB-231 cells after 24 hrs of treatment with E (empty-NP), 25 nM P (paclitaxel-NP), 1.25 µM V (verteporfin-NP), 25 nM C (combretastatin-NP), and different combinations. Data are expressed as mean ± SD, *n* = 3. *P < 0.05, **P < 0.01.

To determine the effect of the three drugs on angiogenetic activity in human TNBC cells, an in vitro luciferase assay was carried out to measure the activity of hypoxia-inducible factor 1-alpha (HIF-1α), a key transcription factor controlling angiogenic genes, tumorigenesis, and stemness factors^25,26^ (Fig. 4A). We found that HIF-1α activity was suppressed after treatment with verteporfin and the three drugs in combination in TNBC MDA-MB-231 cells (Fig. 4B). Verteporfin was also able to inhibit the HIF-1α activity upregulated by paclitaxel and CA4 (Fig. 4B), and the VEGF gene expression upregulated by paclitaxel in TNBC MDA-MB-231 cells (Fig. 4C). Taken together, these results suggest that the three drugs in combination more effectively inhibit the viability of bulk TNBC cells, diminish CSC enrichment, suppress angiogenesis, and circumvent the weaknesses/side effects of paclitaxel and/or CA4 to enhance the treatment efficacy.

### Combination of verteporfin-NP, paclitaxel-NP, and combretastatin-NP decreases ex vivo viability of PDX organotypic slice cultures

**Fig. 5:**
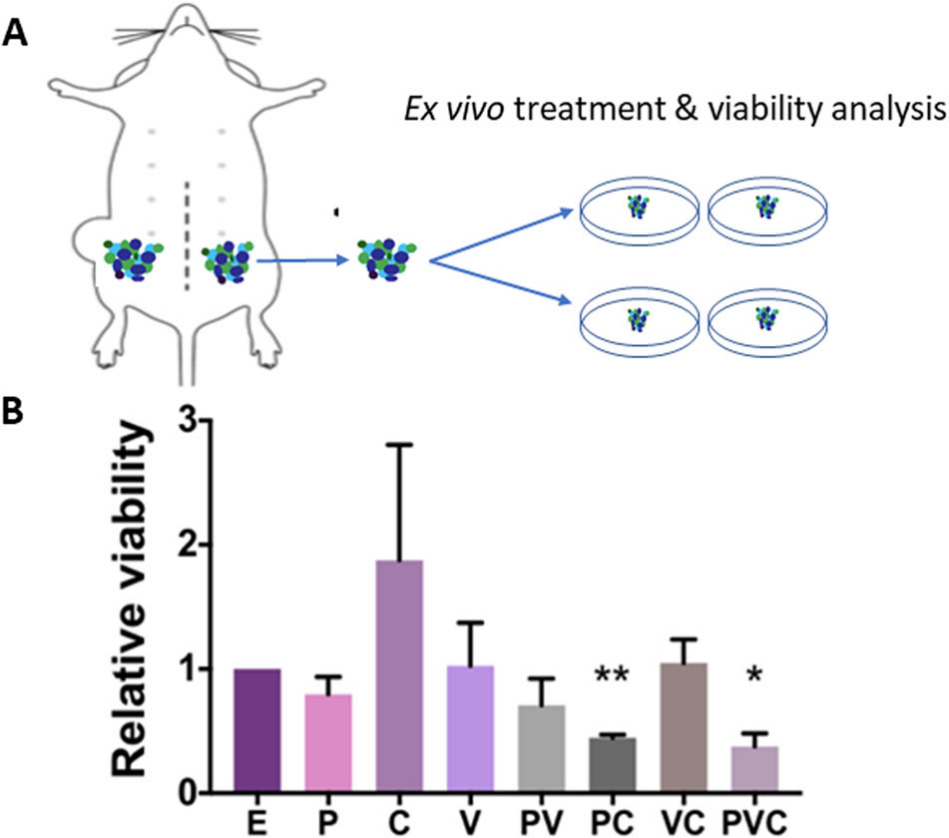
Combination of verteporfin-NP, paclitaxel-NP, and combretastatin-NP decreased ex vivo viability of PDX organotypic slice culture. **A**, **B** Ex vivo viability analysis (Almar blue assay) of PDX (HCI-001) organotypic slice culture after 120-hr treatment with E-NP (empty-NP), P-NP (paclitaxel-NP, 5 nM), V-NP (verteporfin-NP, 250 nM), C-NP (combretastatin-NP, 5 nM), or different combinations. Data represents means ± SEM, *n* = 3, *P < 0.05, **P < 0.01.

To translate our results to a clinical setting, we tested our drug-NPs on PDX organotypic slice cultures. Fragments of organotypic slices were prepared from the PDX tumor and grown ex vivo in a culture dish (Fig. 5A), which has been considered a clinically relevant model^15^. Ex vivo 3D PDX slice cultures contain human tumor cells, stroma cells, extracellular matrices, and other tumor compositions, different from the uniform cancer cell lines cultured in vitro that have gone through a high degree of selection^15^. Because of the limited availability of PDX tumor tissues for in vivo transplantation and substantial experimental logistics, we used organotypic PDX slice cultures that provide a predictable and cost-effective tool for ex vivo selection of drugs for subsequent in vivo PDX transplantation experiments^15^. We surgically engrafted TNBC PDX tumors into athymic mice, passaged and expanded them two to three times in mice. After that, we dissected the tumors, cultured tumor slices, and carried out an Almar blue viability assay after a 120-hr treatment with single drug-NP or the different combinations. The results showed that only the combination of triple drug-NP, or the combination of paclitaxel-NP and CA4-NP achieved a two-fold decrease in tumor slice viability (Fig. 5B). Since the combination of three drug-NPs also inhibited tumorsphere formation and enhanced CSC apoptosis (Fig. 1 and Fig. S4), co-treatment with three drug-NPs seems to be the most effective approach.

### Three drugs co-encapsulated in one NP stopped the growth of TNBC PDX tumor in vivo

While free drugs effectively suppressed MDA-MB-231 cells in vitro (Fig. S7A), they did not delay the growth of MDA-MB-231 tumors in vivo when the mice were treated with free paclitaxel and verteporfin in combination. In contrast, NPs encapsulated with paclitaxel and verteporfin delayed the growth of MDA-MB-231 tumors in vivo (Fig. S7B). In addition, there were no body weight loss and no signs of toxicity observed after 20 days of treatment with NPs encapsulated with drugs (Fig. S5B), suggesting the tolerability of combinational treatment with drugs-NPs in mice. These results provide a rationale for testing drugs-NPs in more clinically relevant PDX models.

**Fig. 6:**
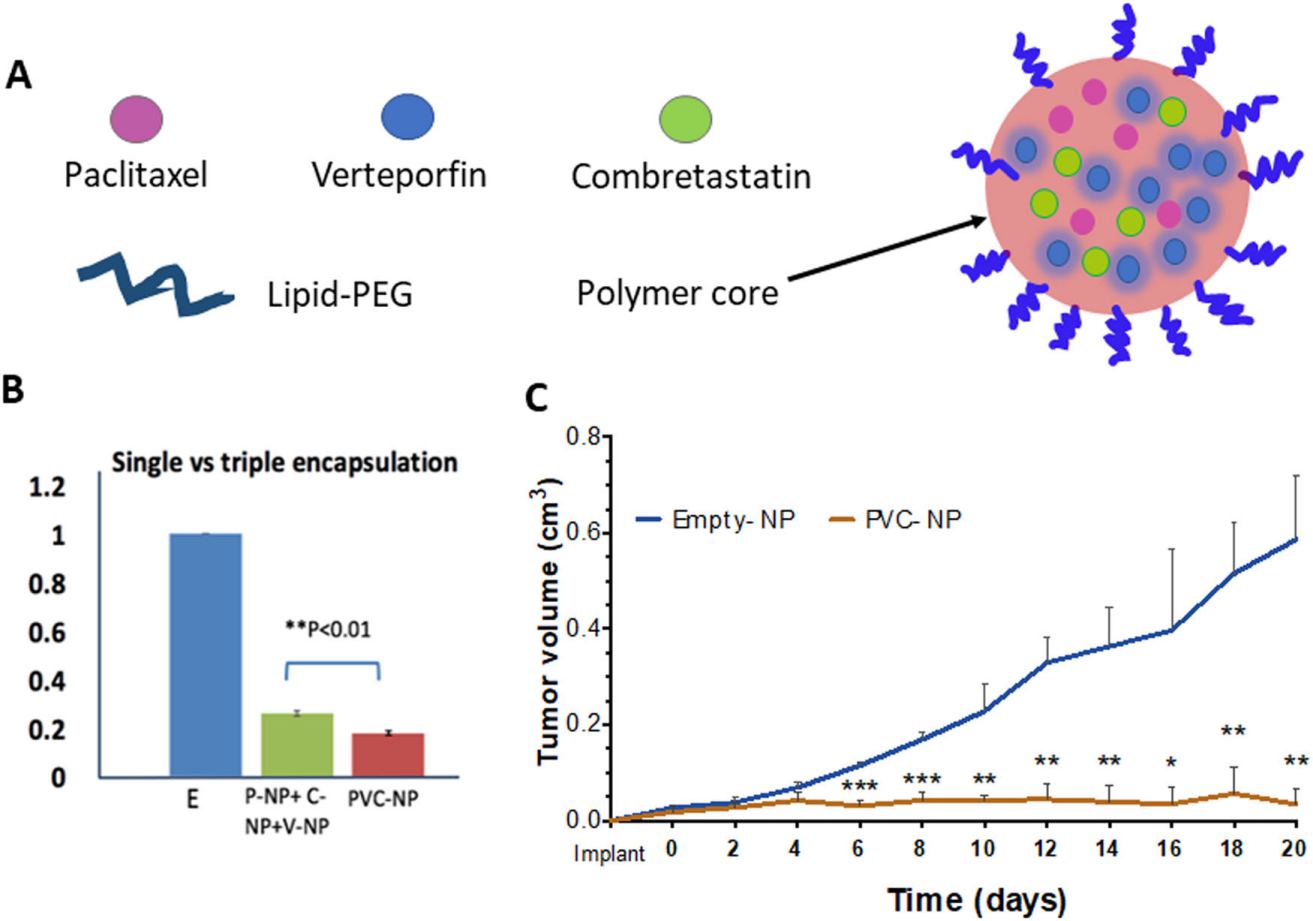
Triple drugs co-encapsulated in one NP stopped TNBC PDX tumor growth in vivo. **A** Schematic showing the development of a triple-drug NP system co-encapsulating CA4, paclitaxel, and verteporfin. **B** Viability analysis using an MTT assay after 120-hr treatment with empty-NP (E), paclitaxel-NP (P-NP) + verteporfin-NP (V-NP) + combretastatin-NP (C-NP), or PVC-NP at a 1:2:1 ratio. Drug-NPs were added at 0 and 72 hr. Three drugs co-encapsulated into NP showed better efficacy than combination of individual drug-NPs. **C** PDX (HCI-002) tumor fragments were engrafted into the mammary fat pads of athymic mice and treated with either control (empty-NP), or PVC-NP (1 mg/kg of paclitaxel and 2 mg/kg verteporfin, 1 mg/kg CA4 co-encapsulated in NP) every 2 days for 20 days. Data are expressed as mean ± SEM, *n* = 3, *P < 0.05, **P < 0.01, ***P < 0.001.

We then asked whether co-encapsulation of three drugs would be more effective than combination of individual drug-NPs. We encapsulated all three drugs into single NPs (Fig. 6A and Fig. S1). As shown in Fig. S1, encapsulation of all three drugs in single NP did not significantly increase the NP size neither did it affect its stability. However, three-drugs co-encapsulated in one NP was more effective than the combination of three separate single drug-NPs at inhibiting the viability of TNBC cell lines (both SUM149 and MDA-MB-231) in vitro, as well as the viability of PDX slice cultures ex vivo (Fig. 6B and Fig. S3).

We then surgically engrafted TNBC PDX tumors^27^ into athymic mice. When the tumors reached a mean diameter of 3 mm, mice were randomized and treated with control E-NPs (empty NPs), or triple drug in one NP (NP co-encapsulated with 1 mg/kg of paclitaxel, 2 mg/kg verteporfin, and 1 mg/kg of combretastatin), every 2 days for 20 days via tail vein injection. The treatment significantly halted PDX tumor growth (Fig. 6C), confirming the efficacy of triple drug in one NP in an in vivo transplantation model. Of note, by the end of the treatment, there were no body weight loss in the mice carrying PDX tumors (mouse body weight: empty-NP 24.16 ± 2.64 g vs PVC-NP 26.40 ± 0.20 g) and no signs of toxicity observed.

## Discussion

**Fig. 7:**
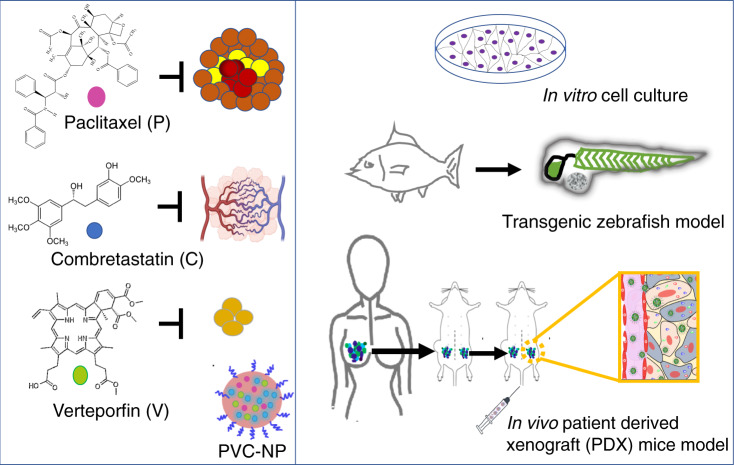
A schematic representation of experimental design using cell culture, transgenic zebrafish, and patient-derived xenograft models to study bulk cancer cells, angiogenesis, and CSCs. PDX: established TNBC PDX fragments were isolated from mice, and engrafted in mice again for in vivo transplantation and for in vitro organotypic slice culture. Biorender was used to construct part of the figure.

TNBC is one of the most aggressive subtypes of breast cancers and accounts for the majority breast cancer-related death due to the lack of a specific target for effective treatment^2^. Targeting CSCs or angiogenesis has been considered effective therapeutic strategies^8,17^. However, chemotherapy enriches CSCs and the disorganized structure of tumor neovasculature impedes free drug delivery and accumulation in the tumors^8,13^. We attempted to circumvent the aforementioned limitations by the development of a three-in-one nanotherapy to inhibit bulk cancer cell, CSCs, and angiogenesis simultaneously (Fig. 7). Individual free drugs selected in this study have been commonly and safely used in patient treatment. The side effects could be further reduced with NP encapsulation, as drugs-NPs have been shown to accumulate more in the tumor^14^.

Nanomedicine has greatly improved the efficacy of drug/small-molecule delivery to the tumor, while minimizing drug off-target toxicities. In this study, we developed and characterized PLGA-PEG NPs encapsulating drugs individually and in combination. The NPs were stable in plasma with NP sizes ~100 nm, which is considered optimal for longer blood circulation and higher accumulation in tumor due to the EPR effect^14,28,29^.

Consistent with existing literature and our previous studies^30–32^, paclitaxel-NP effectively suppresses bulk cells but enriches CSCs that have accounted for tumor recurrence after chemotherapy withdrawal in clinic (Fig. 1 and Fig. S4). We also found that CA4-NP exhibits certain characteristics similar to paclitaxel-NP: inhibiting bulk cancer cell but enriching CSCs, and upregulating CSC-related YAP signal. A possible explanation for this stems from the mechanism of action of CA4. CA4, as an antimitotic agent, acts by binding at the interface of the αβ tubulin heterodimers to inhibit the assembly of microtubules^33^. Recently, a lower expression of αβ tubulin has been associated with a lower degree of differentiation but a higher level of expression of “stemness”/CSC markers^34^. It is possible that CSCs adopt resistance to CA4 by interrupting tubulin heterodimers and microtubule assembling.

Our results also showed that CA4 upregulates YAP target genes, and YAP activation has been associated with anti-tubulin drug resistance^35^. However, the exact mechanistic links between CA4, YAP, and tubulin isotype expression and microtubule assembling remain unclear, warranting further investigations. Of note, verteporfin-NP was able to offset the paclitaxel-NP and CA4-NP-mediated CSC enrichment, highlighting the necessity for combination therapy. Our results also showed that verteporfin-NP effectively decreased cell migration. The inhibitory effect of verteporfin-NP on cancer cell migration and CSCs might be partially attributed to its suppression of YAP activity, which is known to promote EMT/migration and CSCs. The inhibitory effect of verteporfin on CSC enrichment is crucial as suppression of CSCs is well known to prevent tumor recurrence^3,4^. This further highlights the requirement of verteporfin in addition to CA4 and paclitaxel in devising an effective treatment for TNBC to reduce relapse.

While CA4 or verteporfin has been suggested to exhibit antiangiogenic potential separately^9,36^, we showed that combination of two drugs was more effective to suppress angiogenesis in an in vivo transgenic zebrafish model (Fig. 3). Our results also suggest that CA4 exerting its antiangiogenic effect might be partially through the inhibition of Wnt pathway (Fig. S6D). The Wnt pathway is crucial in the functioning of vascular endothelial cells. Wnt signaling controls endothelial cell proliferation, survival, and migration through transcriptional regulation of VEGF^37^. However, the exact mechanism by which CA4 interacts with the Wnt pathway remains a subject for further investigation.

In addition, we found that verteporfin also decreased the activity of HIF-1α and the gene expression of VEGF in human TNBC cells. HIF-1α is a transcriptional factor regulating a wide range of genes involved in angiogenesis (e.g., VEGF), tumorigenesis, and metabolic adaptation of cancer cells, and is commonly linked to poor prognosis in the clinic^26^. We showed that verteporfin was able to subdue paclitaxel-enhanced HIF-1α activity. Paclitaxel-induced HIF-1α activity has been reported to promote breast CSC enrichment and increase the expression of multidrug resistance^38^. Of note, verteporfin has been shown to inhibit HIF-1α DNA-binding ability and HIF-1α-YAP-binding capacity in a liver cancer model^39^. Verteporfin may also exert antiangiogenic effect in TNBC via YAP and/or HIF-1α by counteracting paclitaxel/CA4-upregulated HIF-1α and/or YAP activities. Taken together, our results show that paclitaxel and CA4 are important to decrease the viability of bulk cancer cells, CA4 and verteporfin in combination significantly suppress angiogenesis, and verteporfin reduces CSC enrichment following paclitaxel and CA4 treatment. Therefore, combination of three drugs will improve the outcomes. Of note, those drugs have been individually and safely used in clinic, and combinations of two or three drugs did not further inhibit MCF-10A viability compared to single drug in our experiments (Fig. S5A); no body weight loss and no toxicity were observed in mice after the treatment with E-NPs vs PVC-NPs. These results indicate a possible tolerability of this treatment.

Using an appropriate model in cancer research is crucial for potential clinical translation. PDX organotypic slice cultures and PDX in vivo transplantation have been recognized, as clinically relevant platforms^40^. Various studies have reported that treatment of ex vivo cultured PDX tumor slices correlates with in vivo PDX results, allowing for a more feasible and efficient drug screening^40^. Accordingly, we screened the efficacy of different drug-NPs using ex vivo PDX organotypic slice cultures first, and then assessed the most potential drug-NP in an in vivo PDX model. This has circumvented cost and time consumption, as well as a number of logistic challenges encountered in vivo PDX transplantation.

Using TNBC cell line, in vivo zebrafish model, and clinically relevant PDX models, this study showed the efficacy of a new triple-drug NP system made of FDA-approved biomaterials in TNBC tumors, moving one-step further toward clinical application.

## Materials and methods

Detailed in Supplemental Materials and Methods.

## Supplementary information

Supplemental Materials and Methods

Supplemental Figure Legends

Supplemental Fig S1

Supplemental Fig S2

Supplemental Fig S3

Supplemental Fig S4

Supplemental Fig S5

Supplemental Fig S6

Supplemental Fig S7

Supplemental Table 1
