# Supplementary material for: A triple-drug nanotherapy to target breast cancer cells, cancer stem cells, and tumor vasculature
Source: Cell Death Dis. 2021 Jan 4;12(1):8. doi: 10.1038/s41419-020-03308-w (PMC7791049; doi:10.1038/s41419-020-03308-w)
Supplement: Supplementary file 1 — Supplemental Materials and Methods [file 41419_2020_3308_MOESM1_ESM.docx]

**Supplemental Materials and Methods**

**A triple-drug nanotherapy to target breast cancer cells, cancer stem cells and tumor vasculature**

Sara El-Sahli ^a^, Khang Hua ^b†^, Andrew Sulaiman ^a†^, Jason Chambers ^a^, Li Li ^a^, Eliya Farah ^a^, Sarah McGarry ^a^, Dan Liu ^a,c^, Peiyong Zheng ^d^, Seung-Hwan Lee ^a^, Jiefeng Cui ^e^, Marc Ekker ^b^, Marceline Cote ^a^, Tommy Alain ^a^, Xuguang Li ^f^, Vanessa M. D’Costa ^a,h^, Lisheng Wang ^a,g,h^*, and Suresh Gadde ^a^*

**Materials & Methods**

**Nanoparticles synthesis and characterization**

Lipid-polymer hybrid nanoparticles (NPs) were made through a nanoprecipitation method as previously reported^1^. Lecithin and DSPE-PEG_2K_ in the molar ratio of 6.5:1 were dissolved in 4% ethanol aqueous solution (0.02% w/v) and heated for 2 mins at 68 ^o^C while maintaining gently stirring. PLGA (poly lactic co-glycolic acid) and the selected drug/drugs (10:1 w/w ratio) in either acetonitrile (ACN) were then added dropwise at 0.6ml/min rate while stirring at room temperature to allow the formation of self-assembled hybrid NPs. After 6 hours of stirring, NPs were concentrated and purified by centrifugal filters and characterized for size, surface charge and structure by ZetaView, Malvern Zetasizer (DLS) and TEM. To test the stability of NPs in biologically relevant conditions, NPs were incubated in 5% FBS for 4 hours and size was measured using ZetaView.

**Assessment of drug release of paclitaxel, verteporfin and combretastatin in 72 hours.**

The amounts of paclitaxel and combretastatin remaining in the NPs at different time points were assessed using HPLC at 204 nm, with H2O:acetonitrile mobile phase and with 5%–90% acetonitrile gradient. The amount of verteporfin remaining in the NPs at the different time points was quantified using NanoDrop at 430 nm absorbance. The amounts of drugs calculated were then expressed as percentage of drug released from the NPs.

**Cell culture**

## MDA-MB-231 cell line was purchased from the American Type Culture Collection (Manassas, VA, USA). MDA-MB-231 cells were transfected with pLVX-Tet-On Advanced and pLVX-Tight-Puro MDA-MB-231 cells overexpressing E-cadherin were generated using pLVX-Tight-Puro containing an E-cadherin gene insert according to manufacturer’s instructions (Clontech, Mountain View, California, United States). MDA-MB-231 cells without overexpression of E-cadherin (control) were generated using an empty vector of pLVX-Tight-Puro. Stable clones were selected after 3 days using G418 (Clontech) and puromycin dihydrochloride (Thermo Fisher) at a concentration of 1000 µg/mL and 1 µg/mL respectively for 14 days. For maintenance, 250 µg/mL of G418 and 0.25 µg/mL of puromycin were added in the culture medium. E-cadherin expression was activated by adding 1 µg/mL doxycycline hydrochloride (Thermo Fisher) to the cell culture every 2-3 days. E-cadherin levels were examined following RNA extraction by RT-qPCR and protein levels by western blotting. MDA-MB-231 cells were grown in DMEM-F12 media that contained 10% fetal bovine serum (HyClone, Logan, UT, USA) and 1% penicillin/streptomycin. For the tumorspheres formation assays, E-cadherin high MD-MB-231 cells were seeded in low attachment plates and cultured in serum-free conditions (DMEM/F12 medium containing 1mM sodium pyruvate, 1×B27, 20 ng/mL bFGF and 20 ng/mL EGF).

SUM149 breast cancer cells were obtained from Asterand (Detroit, MI, USA) and cultured in Hams F-12 media (Mediatech, Manassas, VA, USA) containing 5% FBS, 5 μg/ml insulin, 1 μg/ml hydrocortisone, 10 mM HEPES and 1% penicillin/streptomycin. Cells were cultured at 37°C in a 5% CO2 incubator. Insulin, Hydrocortisone, HEPES, and bovine serum albumin were purchased from Sigma-Aldrich (St. Louis, MO, USA).

MCF10A cells (an immortalized, non-transformed epithelial cell line derived from human mammary tissue) were obtained from ATCC. They were cultured in DMEM:F12 (1:1) medium supplemented with 1× antibiotics (penicillin and streptomycin), EGF (10 ng/mL), insulin (10 µg/mL), cholera toxin (1 µg/mL), hydrocortisone (1 µg/mL), and heat-inactivated horse serum (5%) (Invitrogen). All cell lines were incubated at 37 °C in a humidified 5% CO2 atmosphere.

All cell lines used have been recently ruled out for mycoplasma contamination using RT-qPCR.

**Cell viability assay**

MTT stock was diluted in PBS to make 5 mg/mL, aliquoted and stored in -20 °C. Cells were seeded into a 24 well plate (1.5 x 10^3^ cells per well) and treated for 120 hours (unless otherwise stated). MTT was then added to the media in a 1:20 ratio and incubated for 3 hours at 37 °C. Reaction was terminated by aspirating the media and adding 600 µL DMSO to each well. The wells were incubated for 20 min at 37 °C. Triplicates from each well/treatment were aliquoted into a 96 well plate and absorbance at 570 nm was measured using Gen 3.0. Alamar blue assay was performed according to the manufactures’ instructions (Thermo Fisher, Waltham, Massachusetts, United States).

**Cell migration assay**

MDA-MB 231 cells were seeded in 6-well plates and grown to confluence; they were then scratched with a sterile pipette tip, washed with PBS to remove detached cells and debris, and then incubated with 2% mitomycin for 4 hours and then washed with PBS. Cells were then incubated with various treatments for 48 hrs. The scratch was then photographed at different time points using a Zeiss Axiovert 40 CFL microscope (Carl Zeiss AG, Feldbach, Switzerland). The open area was quantified on each photo to obtain a diameter and normalized to that of 0 hours.

**Luciferase Assay to assess HIF-1α activity**

MDA-MB-231 TNBC cells were seeded into 12-well plates and transfected with 1000 ng of HRE-luciferase (HIF-1α, Plasmid #26731, a gift from Dr. Navdeep Chandel [^1^](#_ENREF_1)) construct in conjunction with 1000 ng Renilla pRL-SV40P (Addgene Plasmid #27163, a gift from Dr. Ron Prywes [^2^](#_ENREF_2)) construct using Lipofectamine 3000 (Invitrogen, Carlsbad, California, United States) according to the manufacturer’s instructions. After 18 hours, cells were treated with control and different drugs as described in the Figure legends for 24 hours, after which cells were lysed and both Firefly and Renilla luciferase activities were quantified using a Dual-Luciferase® Reporter Assay System (Promega, Madison, Wisconsin, United States) following the manufacturer's instructions.

**Flow cytometry analysis**

Dissociated cancer cells were filtered through a 4 µm strainer and suspended in PBS supplemented with 2% FBS and 2 mM EDTA (FACS buffer). One µL of mouse IgG (1 mg/mL) was added and incubated at 4 °C for 10 minutes. The cells were then re-suspended in 1× binding buffer and anti-CD44 (APC) in combination with anti-CD24 (PE) antibodies (BD, Mississauga, ON, Canada) according to the manufacturer’s instructions for 30 min. The cells were washed twice with FACS buffer and 7-aminoactinomycin D (7-AAD, eBioscience, San Diego, CA) and Annexin-V/V450 (BD) was added and incubated for 15 minutes at room temperature to assess dead and apoptotic cells. Flow cytometry was performed on the BD LSRFortessa. Data were analyzed with FlowJo software (Ashland, OR, USA).

**RT-qPCR**

To assess mRNA levels, total RNA was extracted from the frozen pellets of MDA MB- 231 using the RNeasy mini kit (Qiagen, Germantown, MD, USA). Nanodrop 1000 (Thermo Fisher) was used to determine mRNA concentration and purity. cDNA was obtained from the mRNA using iScript cDNA Synthesis Kit (Bio-Rad, Hercules, California, United States) and GeneAmp PCR System 2700 (R&D Systems, Minneapolis, Minnesota, United States). Gene expression levels were determined through quantitative real-time PCR (qPCR) analysis using the Bio-Rad MyiQ (Bio-Rad, USA). Reaction mix per well constituted 37.5% RNAase-free H_2_O, 50% SyBr Green (Bio-Rad, USA), 2.5% cDNA and 5% forward and reverse primers of the gene of interest. Reaction conditions were 1 cycle at 95 °C for 20 seconds, 45 cycles at 95 °C for 3 seconds, and 60 °C for 30 seconds. Data obtained were normalized using the housekeeping gene, 18S or GAPDH and relative fold changes of gene expression were calculated via the ^2DD^CT method and determined by comparing it to the appropriate controls. Table S1 contains the primer sequences used.

**Zebrafish treatment and visualization**

Animal care and handling: all zebrafish husbandry and experimental procedures used in the experiments were compliance with University of Ottawa Animal Care Committee following guidelines of the Canadian Council on Animal Care. Transgenic line Tg(*fli*:eGFP) was used for this study and maintained at 28.5^0^C, unless stated otherwise and staged as described earlier^2^.

For *in vivo* zebrafish drug treatment, transgenic Tg(fli:eGFP) embryos were dechorionated at 6 hours-post-fertilization (hpf) using 10 mg/mL Pronase (Sigma-Aldrich) and treated with paclitaxel, verteporfin, combretastatin or various combinations at a final concentration of 5 nM, 250 nM and 5 nM respectively at 8 hpf in 6-well plates with 10 embryos per well and incubated at room temperature in the dark for 48 hours. Solutions were refreshed daily.

**Zebrafish RT-qPCR**

RNA was extracted from pools of 5 whole zebrafish larvae following drug treatment using pestle for homogenization in TRIzol reagent (Invitrogen, Carlsbad, California, United States) following manufacturer protocol. Integrity of RNA was checked by gel electrophoresis, and purity of RNA was determined using the NanoDrop 1000 Spectrophotometer (Thermo Fisher, Waltham, Massachusetts, United States). Only samples with clear 28S and 18S rRNA bands at an approximate intensity ratio of 2:1 and an A260/280 absorbance ratio of 1.8-2.1 were used for cDNA synthesis using the iScript™ Reverse Transcription Supermix for RT-qPCR kit (Bio-Rad) according to the manufacturer protocol. For all genes, standard 10 µL RT-qPCR reactions were assembled as follows: 5 μl of SsoFast™ EvaGreen® Supermix (Bio-Rad), 0.4 μl of forward primer (10 μM), 0.4 μl of reverse primer (10μM), 4 μl of template cDNA and 0.2 μl of nuclease-free water. For no template negative controls, template cDNA was replaced by nuclease-free water. All reactions were carried out in triplicate using the Bio-Rad CFX96 system. The PCR conditions were 95 ºC for 30 seconds, followed by 40 cycles of 95 ºC for 5 seconds and 59 ºC for 5 seconds. Normalized gene expression values were determined using the comparative Cq method and each gene was normalized against three reference genes, *elongation factor 1 alpha* (*ef1α*), *ribosomal protein l13a* (*rpl13a*), and *tyrosine 3-monooxygenase/tryptophan 5-monooxygenase activation protein, zeta polypeptide* (*ywhaz*). All the primers are listed in Table S1.

**Treatment of TNBC MDA-MB-231 tumors growing in mice**

All mouse studies illustrated in this paper were performed according to the ethical guidelines set by the University of Ottawa and in pathogen free conditions. Athymic nude mice were obtained from Charles River Laboratories (Senneville, QC, Canada). The MDA-MB-231 breast cancer cells were mixed 1:1 with Matrigel and injected under aseptic conditions into the mammary fat pads (n = 5 for each group, 2×10^6^ cells per fat pad). When the tumor reached a mean diameter of ~3 mm, mice were injected with empty–nanoparticles, free drugs or same concentrations of drugs-encapsulated in nanoparticles *via* tail vein every 2-day.

**Assessment of *ex vivo* viability of PDX organotypic slice cultures and *in vivo* tumor growth of PDX transplants after treatments**

All protocols described throughout this manuscript regarding animal studies were performed

in strict pathogen-free conditions and in accordance with guidelines approved by the Animal Care Committee at the University of Ottawa. TNBC PDX HCI-002 tumor chunks (2 mm x 4 mm) were transplanted into the mammary fat pad of athymic nude mice (Charles River Laboratories, Senneville, Quebec, Canada). PDX HCI-001 and HCI-002 tumors were originally generated from two patients with TNBC. They have been well characterized, including tumor histology/immunohistology, clinical markers, drug treatment and response, whole exome sequencing, RNA sequencing, RPPA analysis, and growth curve, which have been described in details in the spreadsheet of PDXNet and in the supplemental results published elsewhere [^3^](#_ENREF_3)^,^ [^4^](#_ENREF_4).

For the *ex vivo* organotypic slice culture experiment, HCI-001 tumors were harvested and fragmented into a 48 well plate. Alamar blue viability assay was preformed *via* incubation with 4% Alamar blue solution (Thermo Fisher Scientific, Waltham, MA, USA) for 4 hours after which florescence at 560 nm excitation and 590 nm emission was determined to measure baseline viability. Drugs-NPs were then incubated with tumor fragments for 120 hrs and Alamar blue viability assay was conducted again and calculated by subtracting the values of baseline viability in each group, followed by comparison amongst different groups.

For *in vivo* transplantation and treatment, HCI-002 PDX tumors were passaged twice in athymic nude mice without treatment. Treatment began when the tumors grew to a mean diameter of 3 mm. Mice were randomized into 2 cohorts and treated with either vehicle (empty nanoparticles) or lipid-polymer hybrid nanoparticles co-encapsulating paclitaxel, combretastatin and verteporfin (PVC-NP) every 2 days for 20 days (n=3 mice/group). Tumor growth was measured consistently using a caliper and tumor volume was determined using the formula: V=1/2(Tumor Length x Tumor Width^2^).

**Statistical analysis**

Data are represented as means +/- standard deviation (SD) or standard error (SE) without data transformation. For relative comparison, the data were normalized to control group and then compared as indicated in each figure. Statistical tests were performed using Functions of MS Excel Variances. Data distribution was tested by one-way analysis of variance (ANOVA). Wherever appropriate, statistical differences between groups were assessed by unpaired Student’s two tailed t-test (comparison of two groups) and reported as * for p < 0.05, ** for p < 0.01, *** for p < 0.001, and **** for p < 0.0001. Unless otherwise stated, experiments have a minimum of three biological repeats.

**References**

1 Emerling BM, Weinberg F, Liu JL, Mak TW, Chandel NS. PTEN regulates p300-dependent hypoxia-inducible factor 1 transcriptional activity through Forkhead transcription factor 3a (FOXO3a). *Proceedings of the National Academy of Sciences of the United States of America* 2008; **105**:2622-2627.

2 Chen X, Prywes R. Serum-induced expression of the cdc25A gene by relief of E2F-mediated repression. *Molecular and cellular biology* 1999; **19**:4695-4702.

3 PDXNet. PDX DEVELOPMENT AND TRIAL CENTERS RESEARCH NETWORK. *https://brandi-davis-7wsfsquarespacecom/hcibcm*.

4 DeRose YS, Wang G, Lin YC *et al.* Tumor grafts derived from women with breast cancer authentically reflect tumor pathology, growth, metastasis and disease outcomes. *Nat Med* 2011; **17**:1514-1520.
