# Supplementary material for: A triple-drug nanotherapy to target breast cancer cells, cancer stem cells, and tumor vasculature
Source: Cell Death Dis. 2021 Jan 4;12(1):8. doi: 10.1038/s41419-020-03308-w (PMC7791049; doi:10.1038/s41419-020-03308-w)
Supplement: Supplementary file 2 — Supplemental Figure Legends [file 41419_2020_3308_MOESM2_ESM.docx]

**Supplemental Figure Legends**

**A triple-drug nanotherapy to target breast cancer cells, cancer stem cells and tumor vasculature**

Sara El-Sahli ^a^, Khang Hua ^b†^, Andrew Sulaiman ^a†^, Jason Chambers ^a^, Li Li ^a^, Eliya Farah ^a^, Sarah McGarry ^a^, Dan Liu ^a,c^, Peiyong Zheng ^d^, Seung-Hwan Lee ^a^, Jiefeng Cui ^e^, Marc Ekker ^b^, Marceline Cote ^a^, Tommy Alain ^a^, Xuguang Li ^f^, Vanessa M. D’Costa ^a,g^, Lisheng Wang ^a,g,h^*, and Suresh Gadde ^a^*

**Fig. S1. Development of lipid polymer nanoparticles (NPs) encapsulated with paclitaxel, verteporfin and/or combretastatin and their physiochemical properties.** **A.** TEM microscopic image of NP encapsulated with three drugs (scale bar, 100 nm). **B.** and **C.** Sizes of single and triple encapsulated NPs measured by Dynamic Light Scattering (DLS) before incubation with serum (B) and after incubation with serum (C) for 4 hours. **D.** Zeta potential showing surface charge of single and triple encapsulated NP. **E.** Representative size distribution.

**Fig. S2. Drug release profile of paclitaxel, verteporfin and combretastatin in 72 hours.** The amounts of paclitaxel (Pacli) and combretastatin (CA4) remaining in the NPs at different time points were analyzed using HPLC at 204 nm, with H2O:acetonitrile mobile phase and with 5%–90% acetonitrile gradient. The amounts of verteporfin (Vp) remaining in NPs at the different time points were quantified using NanoDrop at 430 nm absorbance. The amounts of drugs were expressed as percentage of drug released from the NPs.

**Fig. S3. Triple drug-encapsulated NPs are more effective at suppressing TNBC cells and TNBC slice cultures than the combination of single drug-encapsulated NPs. A** and **B.** The viability of SUM149 and MDA-MB-231 TNBC cells was determined by MTT assays after 48-hour treatment. **C.** Viability of PDX (HCI-001) *ex vivo* slice culture assessed by Alamar blue assay after 72-hour treatment, with statistical difference. NP (nanoparticles), E (empty-NP), P-NP (5nM), V-NP (10 nM), C-NP (5 nM), VC-NP (10nM:5nM), PV-NP (5nM:10nM), PC-NP (5nM:5nM), PVC-NP (5nM:10nM:5nM). n = 3 for (**A)** and (**B), n** = 5 for (**C)**. Data represent means ± SEM, * p < 0.05, ** p < 0.01.

**Fig. S4. Verteporfin enhances CSC apoptosis.** Flow cytometric analysis of CD44^+^ CD24^-^ CSCs and Annexin V^+^ MDA-MB-231 cells after 96 hours of treatments with verteporfin, paclitaxel, combretastatin, or in different combinations.

**Fig. S5. The combination of drug-NP treatment does not significantly inhibit non-transformed mammary epithelial cells *in vitro*, and does not cause the loss of mouse body weights *in vivo.* A.** The viability of MCF-10A cells was determined by an MTT assay after 120-hour treatment with E (empty-nanoparticle), P (paclitaxel-NP, 5nM), V (verteporfin-NP, 250 nM), C (combretastatin-NP, 5 nM), or different combinations. **B.** There was no body weight loss observed after treatment with empty-NP (empty-nanoparticles) or nanoparticle encapsulated with drugs for 20 days. N = 3 mice in each group.

**Fig. S6**. **The effect of drug treatment on ZF embryos.**

**A-B.** Representative images of zebrafish embryos after treatment with paclitaxel or paclitaxel + verteporfin, which were not shown in Figure 3. Zebrafish embryos were treated when they were 8 hours post fertilization (hpf) for 48 hours. At 56 hpf, they were imaged for vascular disruption. **C.** Quantitative analysis of A-B. **D.** RT-qPCR analysis of Wnt target gene *axin2* in zebrafish embryos.

**Fig. S7. The effect of free drugs and drugs-NPs on TNBC cells *in vitro* and on MDA-MB-231 tumors *in vivo.* A.** The viability of TNBC MDA-MB-231 cells was determined by MTT assays after 48-hour treatment with D (DMSO), or combination of free drug P (paclitaxel, 5 nM), V (verteporfin, 10 nM), and C (combretastatin, 5 nM). N = 3, **** p < 0.0001 compared to control. **B.** MDA-MB-231 TNBC cells were injected into the mammary fat pads of athymic nude mice (2×10^6^ cells per mammary pad). When the tumors reached a mean diameter of ~3 mm, mice were treated with empty-NP (empty nanoparticles), FD (free drug, paclitaxel 1mg/kg and verteporfin 10mg/kg), or NP (nanoparticles encapsulated with paclitaxel and verteporfin at the same concentration as free drugs). Free drugs did not exhibit inhibitory effect on tumor growth after treatment for 20 days while drug-NP treatment delayed tumor growth. N = 5 mice in each group, * p < 0.05 compared to free drug group or empty-nanoparticle group.
