# Supplementary figures and images for: A triple-drug nanotherapy to target breast cancer cells, cancer stem cells, and tumor vasculature
Source: Cell Death Dis. 2021 Jan 4;12(1):8. doi: 10.1038/s41419-020-03308-w (PMC7791049; doi:10.1038/s41419-020-03308-w)

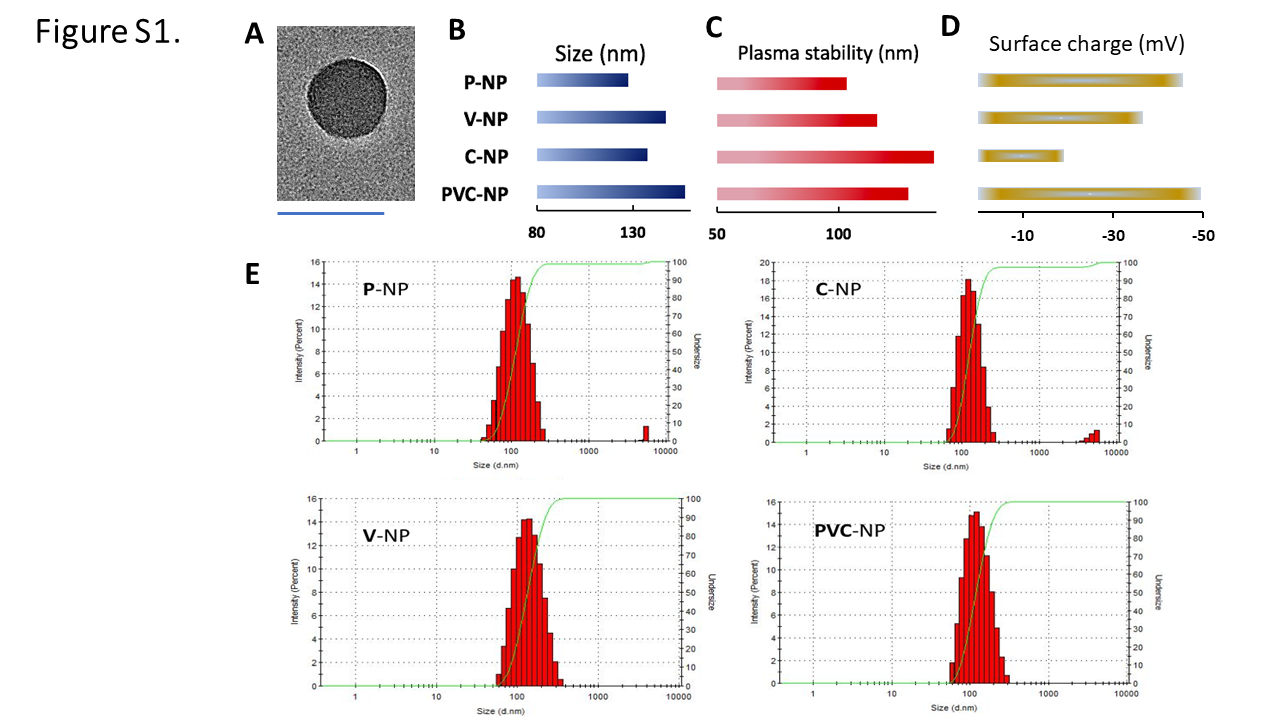

Supplement: Supplementary file 3 — Supplemental Fig S1 [file 41419_2020_3308_MOESM3_ESM.tif]

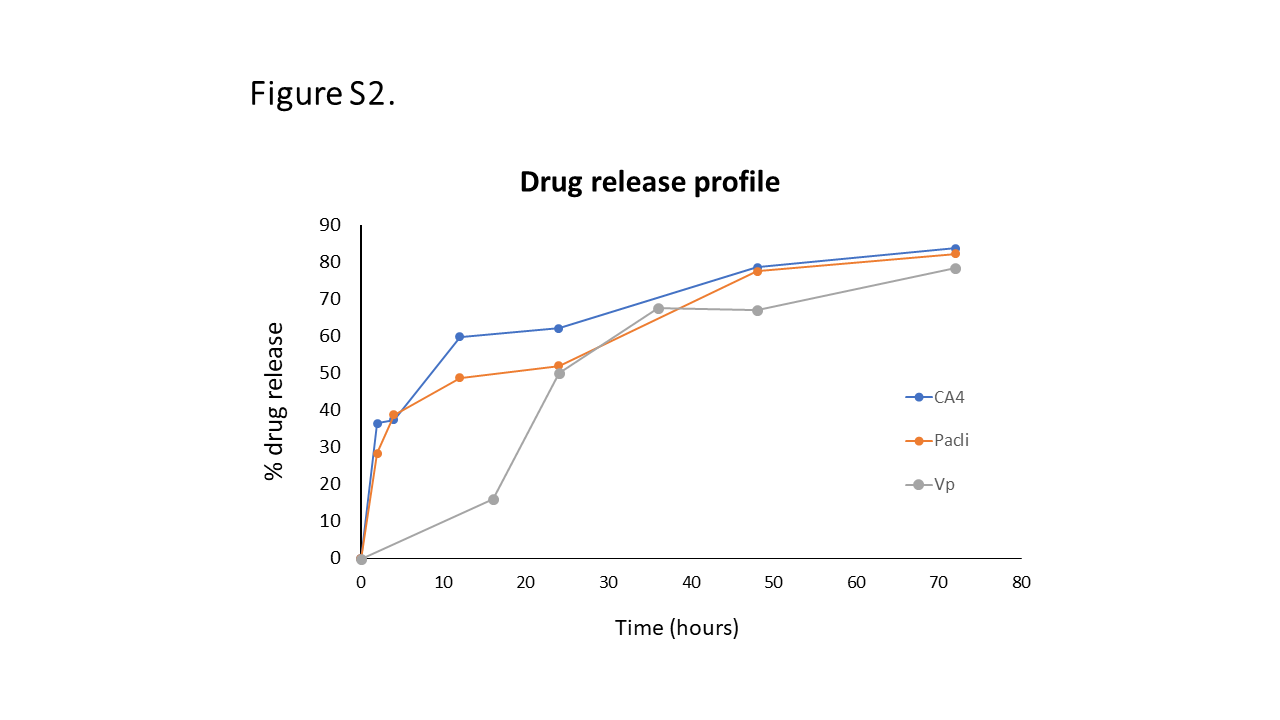

Supplement: Supplementary file 4 — Supplemental Fig S2 [file 41419_2020_3308_MOESM4_ESM.tif]

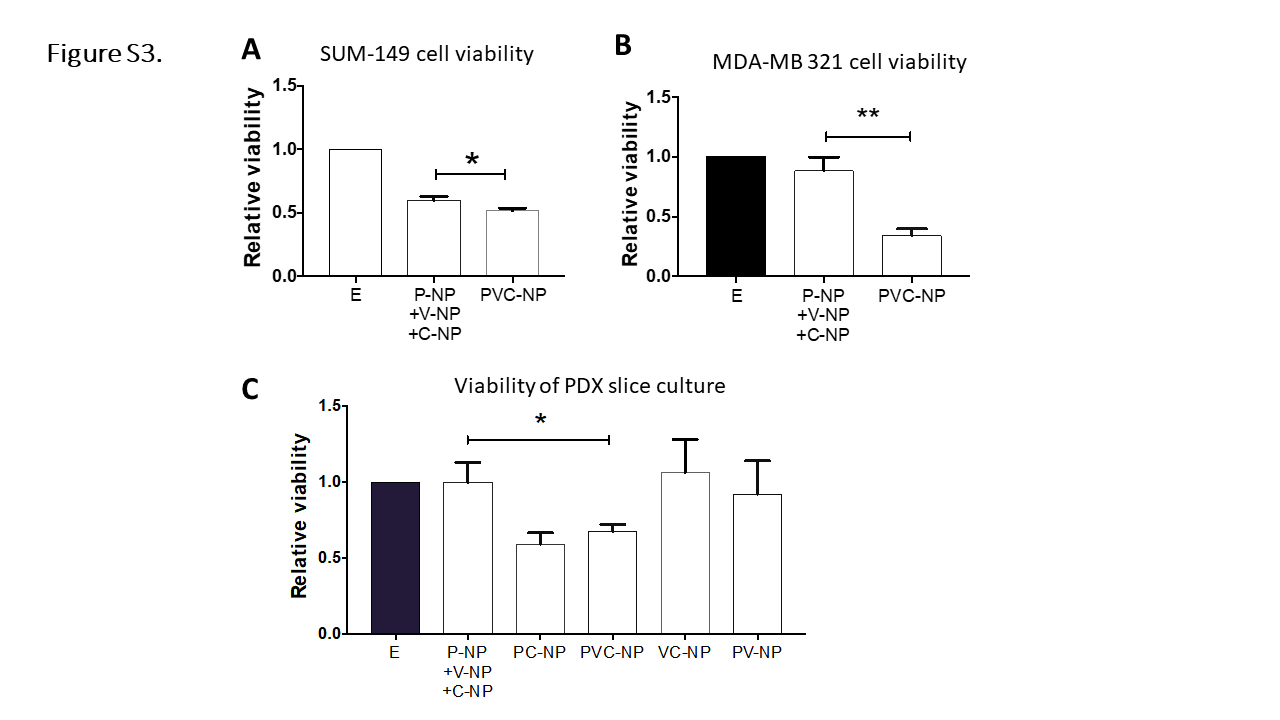

Supplement: Supplementary file 5 — Supplemental Fig S3 [file 41419_2020_3308_MOESM5_ESM.tif]

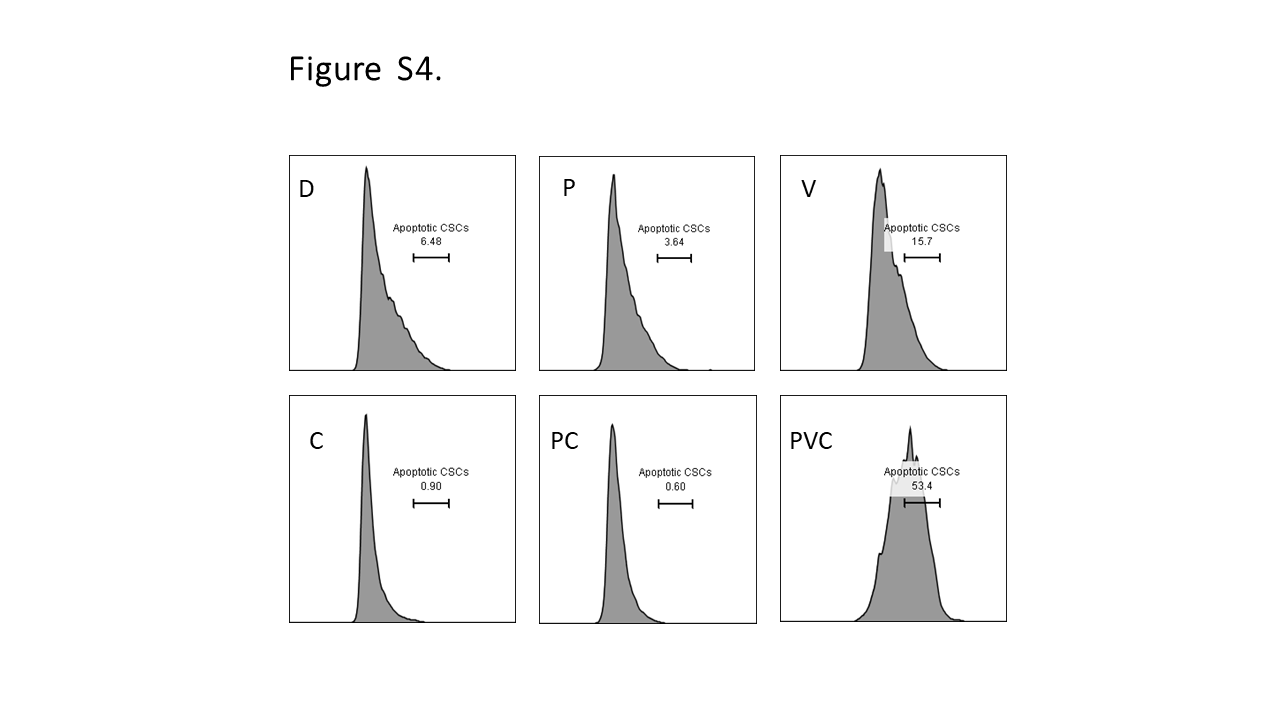

Supplement: Supplementary file 6 — Supplemental Fig S4 [file 41419_2020_3308_MOESM6_ESM.tif]

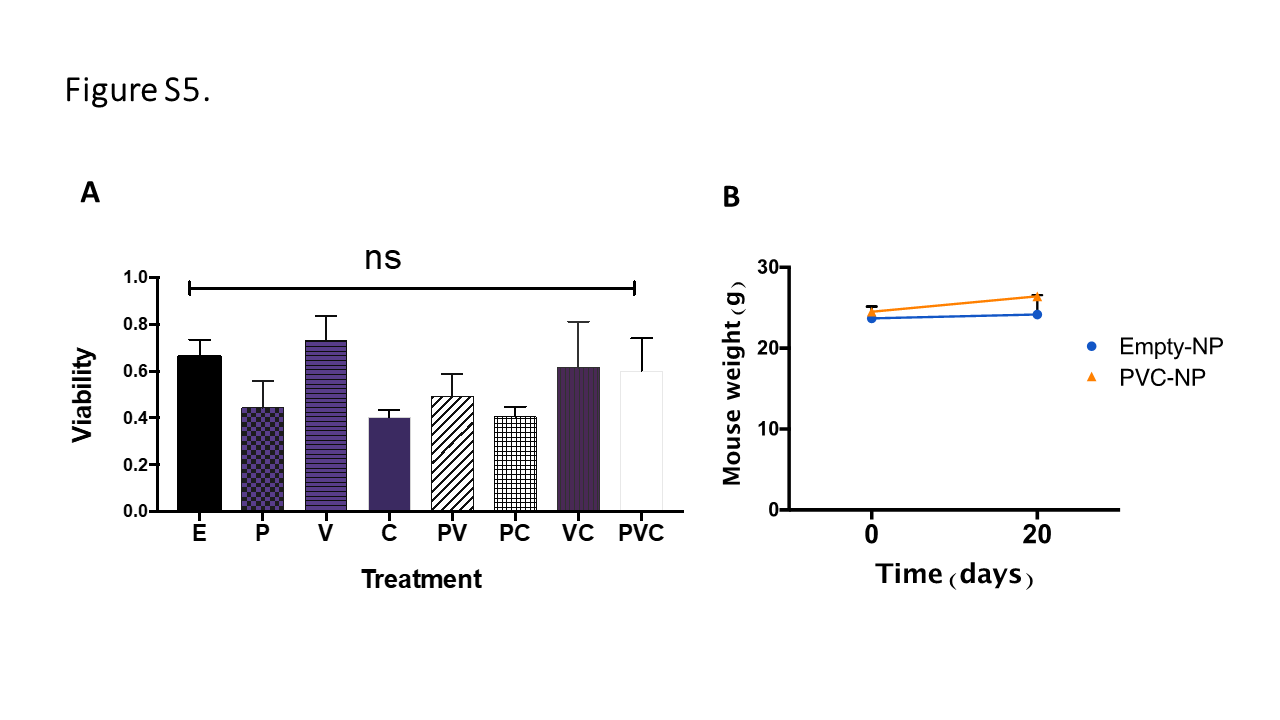

Supplement: Supplementary file 7 — Supplemental Fig S5 [file 41419_2020_3308_MOESM7_ESM.tif]

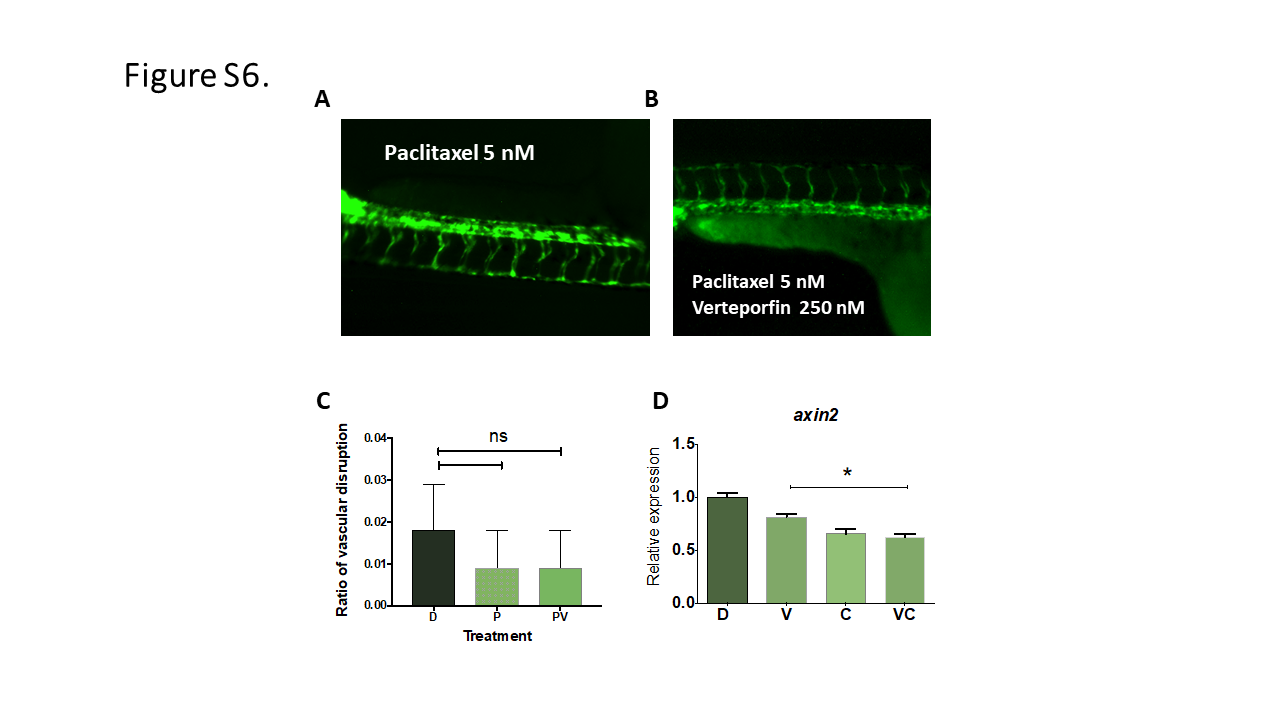

Supplement: Supplementary file 8 — Supplemental Fig S6 [file 41419_2020_3308_MOESM8_ESM.tif]

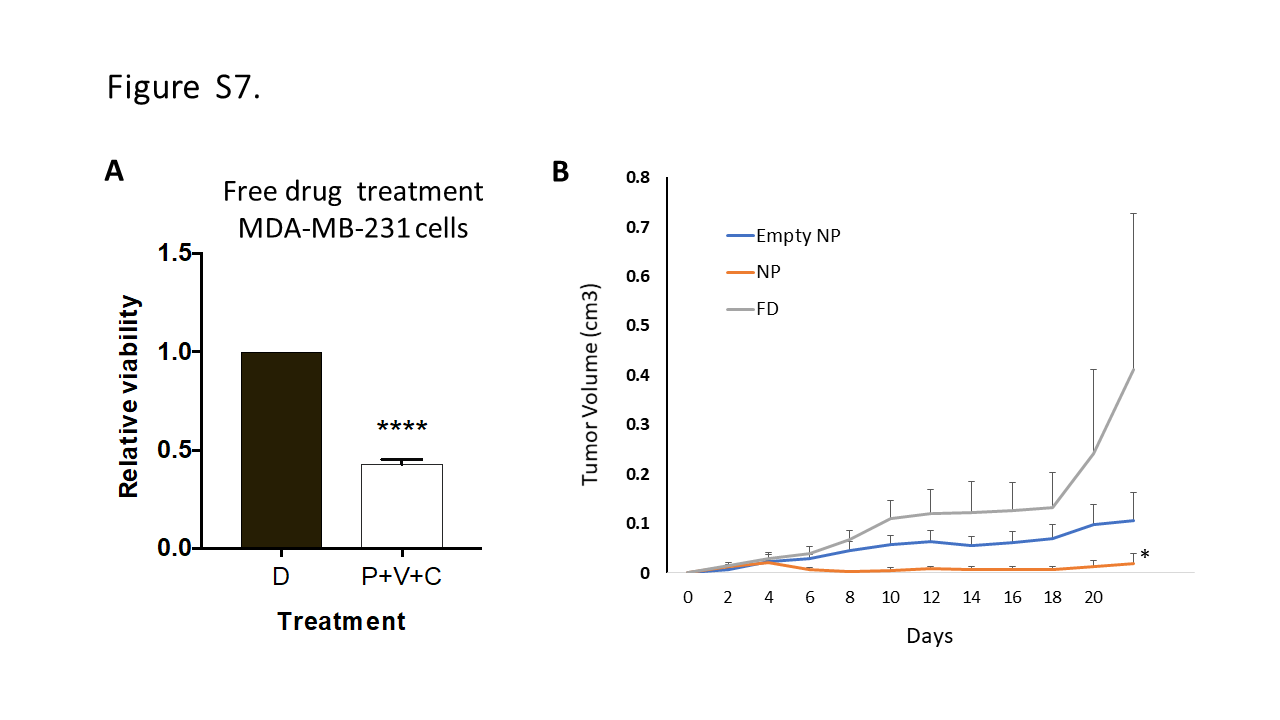

Supplement: Supplementary file 9 — Supplemental Fig S7 [file 41419_2020_3308_MOESM9_ESM.tif]
