# Supplementary material for: A triple-drug nanotherapy to target breast cancer cells, cancer stem cells, and tumor vasculature
Source: Cell Death Dis. 2021 Jan 4;12(1):8. doi: 10.1038/s41419-020-03308-w (PMC7791049; doi:10.1038/s41419-020-03308-w)
Supplement: Supplementary file 10 — Supplemental Table 1 [file 41419_2020_3308_MOESM10_ESM.docx]

**Supplemental Table 1.**

Primers used for the assessment of gene expressions of human TNBC cells by RT-qPCR

| **Primer** | **Forward primer 5’-3’** | **Reverse primer 5’-3’** |
| --- | --- | --- |
| **CTGF** | AGGAGTGGGTGTGTGACGA | CCAGGCAGTTGGCTCTAATC |
| **Cyr61** | AGCCTCGCATCCTATACAACC | TTCTTTCACAAGGCGGCACTC |
| **18S** | AACCCGTTGAACCCCATT | CCATCCAATCGGTAGTAGCG |
| **GAPDH** | ACAGTCAGCCGCATCTTCTT | GACAAGCTTCCCGTTCTCAG |

Primers used for the assessment of gene expressions of zebrafish by RT-qPCR

| **Primer** | **Forward Sequence 5’-3’** | **Reverse Sequence 5’-3’** |
| --- | --- | --- |
| **axin2** | GGACACTTCAAGGAACAACTAC | CCTCATACATTGGCAGAACTG |
| **vegfa** | TCCAGGAGTATCCCGATGAG | GCTTTGACTTCTGCCTTTGG |
| **flt-1** | ATGGGAACAGCAGCACTCTT | TTGAAGACGGAGGGACAATC |
| **kdr** | TGTGGTCAGCTATGCTGGAG | AGCCTCTCATGCTGTGGACT |
| **ef1α** | CTGGAGGCCAGCTCAAACAT | ATCAAGAAGAGTAGTACCGCTAGCATTAC |
| **rpl13a** | TCTGGAGGACTGTAAGAGGTATGC | AGACGCACAATCTTGAGAGCAG |
| **ywhaz** | TCTGCAATGATGTGTTGGAGC | TCAATGGTTGCTTTCTTGTCGTC |
